# The use of early postoperative prostate-specific antigen to stratify risk in patients with positive surgical margins after radical prostatectomy

**DOI:** 10.1186/1471-2490-14-79

**Published:** 2014-10-02

**Authors:** Stepan Vesely, Ladislav Jarolim, Katerina Duskova, Marek Schmidt, Pavel Dusek, Marko Babjuk

**Affiliations:** Department of Urology, Charles University 2nd Faculty of Medicine University Hospital Motol, V Uvalu 84, Prague, 5-150 06 Czech Republic

**Keywords:** Prostate specific antigen, Radical prostatectomy, Surgical margins, Adjuvant radiotherapy

## Abstract

**Background:**

It is well recognized that the presence of positive surgical margins (PSM) after radical prostatectomy (RP) adversely affects cancer specific outcomes and recent evidence from randomized trials supports the use of adjuvant radiotherapy in these cases. However, not all of the patients with PSM develop disease recurrence and the policy of adjuvant radiation could result in considerable over-treatment. We investigated the ability of early postoperative prostate specific antigen (PSA) and PSA decline rates to stratify the risk of disease progression during the first weeks after the surgery thereby allowing adequate time for planning eventual adjuvant therapy.

**Methods:**

We studied 116 consecutive patients with the finding of PSM after RP for localized prostate cancer between 2001 and 2012. No patients were treated with radiation or hormonal therapy. An intensive postoperative PSA monitoring using ultrasensitive assay started first at day 14 after the surgery, then at day 30, 60, 90 and 180, and subsequently in 3 monthly intervals. Biochemical recurrence (BCR) presented the failure of surgical treatment and it was defined as PSA ≥0.2 ng/ml. The ability of PSA decline parameters to predict BCR was assessed using Cox regression model and area under the curve (AUC) calculation.

**Results:**

Overall 55 (47%) patients experienced BCR during median follow-up of 31.4 months (range 6–69). Preoperative PSA, pathologic Gleason sum and pathologic grade failed to reveal any association with observation of BCR. Postoperative PSA levels achieved significant predictive accuracy already on day 30 (AUC 0.74). PSA >0.073 ng/ml at day 30 increased significantly the risk of BCR (HR 4.35, p < 0.001). Predictive accuracy was significantly exceeded on day 60 (AUC 0.84; p < 0.001), while further enhancements on day 90 (AUC 0.84) and 180 (AUC 0.91) were not significant.

**Conclusions:**

The level of ultrasensitive PSA yields valuable information about the prostatectomy outcome already at the first month after the surgery and should aid risk stratification in patients with PSM. Patients not likely to experience subsequent disease progression may be spared the toxicity of immediate adjuvant radiotherapy.

## Background

Although radical prostatectomy provides excellent control for localized prostate cancer, pathologic examination of approximately one-third of specimens will reveal positive surgical margins (PSM) [1, 2]. Numerous studies report that the presence of PSM adversely affects cancer specific outcomes and considerably increases the risk of biochemical recurrence (BCR) [3]. However, the optimum management of patients with PSM remains controversial.

Evidence from randomized trials suggests that immediate radiotherapy after the surgery, rather than watchful waiting, is more appropriate for the patient with pathologically advanced disease because it can improve cancer-specific and overall survival [4–6]. Despite the fact that the most contemporary guidelines do not uniformly recommend adjuvant therapy for patients with adverse pathologic characteristics at radical prostatectomy, it has been shown that in daily clinical practice the patients with PSM were significantly more likely to receive immediate adjuvant treatment [7].

But not all of the patients with PSM develop BCR and the policy of adjuvant radiotherapy could result in considerable over-treatment. Therefore correct identification of those patients most likely to benefit from adjuvant management is of paramount importance. However, attempts to improve early staging after the surgery already hint at several difficulties. The ability of imaging modalities remains limited [8]. The impact of PSM-associated variables (location, focality, length and Gleason score at the margin) on clinical decision making was not clearly established yet [2]. Even routine use of frozen section on all cases has not fulfilled its expectation to provide effective control of surgical margin status [9, 10].

Postoperative PSA measurements are generally performed 3 months after the surgery, although a significant decline in PSA may be detectable much earlier. Results from several studies indicate that intensive monitoring of PSA changes early after radical prostatectomy may provide clinically useful information, which facilitates identification of surgical failure [11–14]. Moreover, recently introduced ultrasensitive PSA detection techniques are offering a new insight into the changes in serum PSA at very low concentration. It has been demonstrated that after a properly performed radical prostatectomy, measurable PSA is most likely attributed to the presence of active prostate cancer cells rather than to retained benign prostatic tissue [15, 16]. It is therefore conceivable, that real candidates for immediate adjuvant therapy, who have active prostate cancer cells remaining in the body after the surgery, should present with higher postoperative serum PSA in a comparison to individuals with incorrect diagnosis of PSM.

This hypothesis prompted us to evaluate the ability of early postoperative ultrasensitive PSA levels to reduce the overtreatment rate by further stratification of potential candidates for immediate adjuvant radiotherapy.

## Methods

The study has received ethical approval by institutional review board of the University Hospital Motol (approval reference: EK-377/13). Data from 871 consecutive patients who underwent open or laparoscopic radical prostatectomy for clinically localized prostate cancer between May 2001 and March 2012 at our institution were reviewed. Pathological evaluation of prostate cancer surgical specimen revealed PSM in 183 patients (21.0%). Of these 183 patients, 63 (34.4%) received adjuvant treatment in terms of radiation or hormonal manipulation and these patients were excluded from the analysis. In order to provide the most accurate calculation of postoperative PSA dynamic, patients treated with neo-adjuvant hormonal and/or radiation therapy prior to the surgery were excluded from the study as well (n = 2). Additionally, since pelvic lymphadenectomy was not routinely performed in all of the patients, nodal involvement was not included in the statistical analysis and these patients (n = 2) were excluded from the study. This resulted in a final cohort of 116 patients available for statistical evaluation. Statistical comparison of clinico-pathological characteristics (PSA at diagnosis, Gleason grade, T stage) of patients with PSM excluded from the study did not differ significantly from the studied cohort (Chi-square test, Mann–Whitney test).

A positive surgical margin was defined as the presence of tumor at the inked surface of the resected specimen. Tumors were staged according to the 2002 TNM staging system. Extraprostatic extension was defined as the extension of the tumor beyond the confines of the gland into the periprostatic soft tissue. Prostate cancer Gleason grading was performed by a dedicated genitourinary pathologist. PSA determinations were carried out postoperatively on days 14, 30, 60, 90, 180 and at three monthly intervals thereafter. All the PSA tests were performed in a single hospital laboratory under standardized settings using the Immulite third-generation PSA assay (Diagnostic Products Corp, Los Angeles, California; lower detection limit 0.003 ng/ml). Biochemical recurrence was defined as a single post-nadir PSA level of 0.2 ng/ml or greater.

Statistical analysis was performed with the SAS statistical software program JMP 6 (SAS Institute, Cary, NC, USA). Mann–Whitney test and Chi-square test were used to compare several variables between groups of patients. The cut-off values of serum PSA that best predicted the biochemical progression were determined by using the Partition platform of the software. Patients were censored at the time of their last tumor-free clinical follow-up appointment. Survival analysis was performed using the Cox proportional hazard model. Pearson's correlation coefficient was used to examine the relationship between continuous variables. The receiver operating characteristic curve (ROC) and area under the curve (AUC) were determined to describe the accuracy in predicting BCR post-surgically. The significance of the difference in predictive accuracy between areas under particular ROC curves was assessed with the method of DeLong et al. [17]. A P value less than or equal to 0.01 was considered statistically significant.

## Results

For a total of 116 patients with the finding of PSM after radical prostatectomy, median follow-up was 31.4 months (range 6–69). Of this cohort 55 (47%) patients experienced BCR. Median patient age at operation was 64 years (range 49–76). Median duration of time to BCR was 12 months (range 2–66). Median preoperative value of PSA was 9.2 ng/ml (range 2.8-38.2). Considering the clinical stage the distribution of the patients was as follows: T1c (n = 64), T2a (n = 28), T2b (14) and T2c (n = 10). The frequency of BCR did not differ significantly (p = 0.08) between clinical T categories: T1c (38%), T2a (54%), T2b (71%) and T2c (60%). Pathological examination of the species revealed Gleason score ≥7 in 59 (51%) patients, extraprostatic extension in 51 (43.9%) patients and seminal vesicle invasion in 11 (9.48%) patients. Except at day 14, postoperative PSA levels were identified to be significantly associated with observation of BCR (P < 0.001), while other conventional clinicopathologic variables failed to reveal significance (Table 1). Of all PSM locations, 15 (13%) were apical, 20 (17%) at the bladder neck and 81 (70%) at the posterolateral site. A total of 46 patients (40%) had PSM ≤ 1 mm. Neither the location (p = 0.216) nor the extent of PSM (p = 0.405) had any significant impact on the frequency of BCR. There were 36 men with the combination of all the adverse pathologic features (PSM and Gleason score ≥7 and extraprostatic extension) and these patients did not experienced significantly different rate of BCR (55%) in the comparison with the rest of the cohort (44%, P = 0.293). Calculated cut-off values for particular PSA measurement on day 14, 30, 60, 90 and 180 were 0.707 ng/ml, 0.073 ng/ml, 0.041 ng/ml, 0.012 ng/ml and 0.021 ng/ml, respectively.

**Table 1:** **Differences in potential predictive parameters according to the appearance of BCR**

Parameters	BCR	BCR-free	***p***value
n	55	61	
Pre-operative PSA (ng/ml, range)	9.20 (2.85-38.20)	7.60 (3.08-59.7)	0.172
Pathologic Gleason sum ≥ 7	27 (49.1%)	32 (52.5%)	0.717
Pathologic extraprostatic extension	25 (45.5%)	26 (42.6%)	0.393
Seminal vesicle invasion	8 (14.5%)	3 (5%)	0.077
PSA – day 14 (ng/ml, range)	0.298 (0.071-1.940)	0.238 (0.045-3.020)	0.264
PSA – day 30 (ng/ml, range)	0.085 (0.008-0.747)	0.022 (0.003-0.692)	<0.0001
PSA – day 60 (ng/ml, range)	0.041 (0.003-0.882)	0.010 (0.003-0.618)	<0.0001
PSA – day 90 (ng/ml, range)	0.042 (0.003-1.010)	0.008 (0.003-0.853)	<0.0001
PSA – day 180 (ng/ml, range)	0.013 (0.015-1.167)	0.011 (0.003-0.931)	<0.0001
PSA nadir (ng/ml, range)	0.022 (0.003-0.137)	0.007 (0.003-0.039)	<0.0001
Time to PSA nadir (months, range)	2 (0.5-3)	2 (1–12)	0.005

BCR, biochemical recurrence; PSA, prostate specific antigen;

Data presented as n (%) or median (range).

The ability of postoperative PSA values to predict surgical failure was tested in the Cox proportional model. Apart from non-significant result for PSA at day 14, the risk of surgical failure predicted by PSA was increasing gradually with the time distance from the surgery (Table 2). Correlation between the preoperative PSA and postoperative ultrasensitive PSA was significant only on day 14 (r = 0.64, P < 0.001) and day 30 (r = 0.22, P < 0.01), while at day 60 (r = 0.05, P = 0.61), day 90 (r = 0.013, P = 0.89) and day 180 (r = 0.16, P = 0.10) no significance was found.

**Figure 1:**
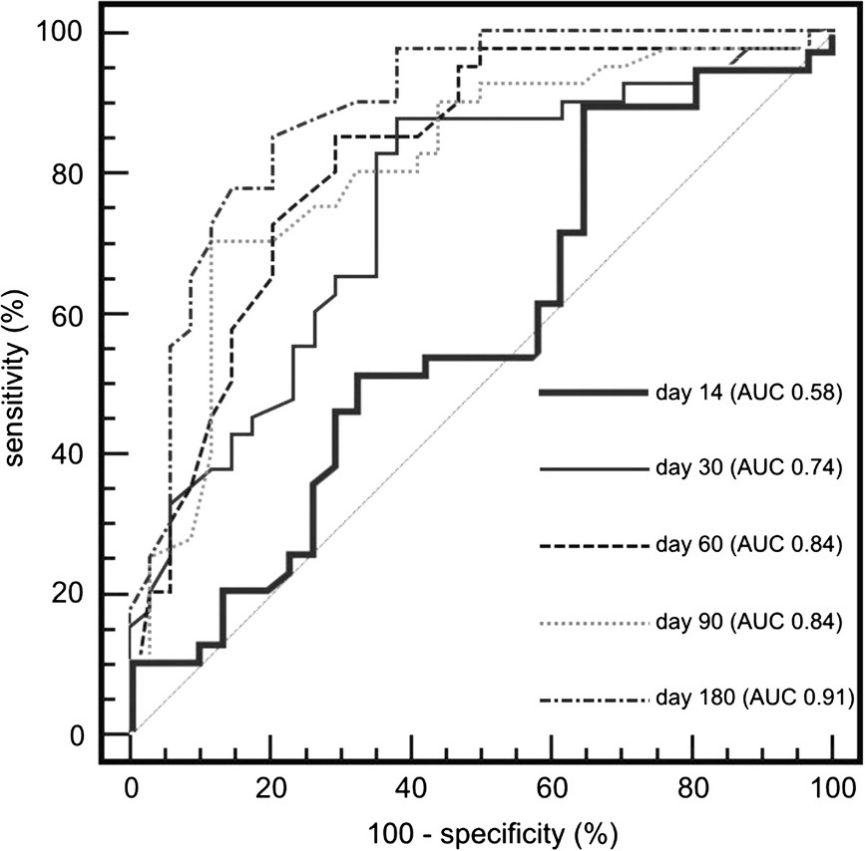
**Receiver operating characteristics (ROC) curves and calculated area under the curve (AUC) values for PSA at particular postoperative measurement day, devised for predicting biochemical recurrence.**

Calculated AUC values for PSA cut-offs on day 14, 30, 60, 90 and 180 were 0.58 (95% CI: 0.45-0.69; P = 0.259), 0.74 (95% CI: 0.64-0.82; P < 0.001), 0.84 (95% CI: 0.75-0.91; P < 0.001), 0.84 (95% CI: 0.75-0.90; P < 0.001) and 0.91 (95% CI: 0.84-0.96; P < 0.001), respectively. ROC curves and calculated AUC values are depicted in Figure 1. Positive/negative predictive values for particular PSA cut-offs on day 14, 30, 60, 90 and 180 were 73%/63%, 81%/72%, 96%/67%, 73%/90% and 83%/87%. Calculated PSA decline adjusted for preoperative baseline (PSA on particular measurement day/preoperative PSA) did not improve the prediction of BCR. Calculated AUC values for PSA decline cut-offs on day 14, 30, 60, 90 and 180 were 0.52 (95% CI: 0.40-0.64; P = 0.745), 0.72 (95% CI: 0.62-0.80; P < 0.001), 0.82 (95% CI: 0.73-0.89; P < 0.001), 0.82 (95% CI: 0.73-0.88; P < 0.001) and 0.88 (95% CI: 0.81-0.94; P < 0.001), respectively.

**Table 2:** **Cox regression analyses of PSA cut-offs at particular postoperative measurement day as a predictor of BCR**

Parameters	Hazard ratio	***p***value
PSA – day 14 (0.707 ng/ml)	2.60 (1.24-5.56)	0.133
PSA – day 30 (0.073 ng/ml)	4.35 (2.39-8.06)	<0.001
PSA – day 60 (0.041 ng/ml)	9.59 (5.15-17.95)	<0.001
PSA – day 90 (0.012 ng/ml)	12.34 (4.98-41.09)	<0.001
PSA – day 180 (0.021 ng/ml)	12.85 (6.29-29.82)	<0.001

BCR, biochemical recurrence; PSA, prostate specific antigen; PSM, positive surgical margins.

Data presented as PSA-measurement day (cut-off value) and hazard ratio (95% confidence interval).

Applying the PSA cut-off at day 30 as the indicator for adjuvant radiotherapy would result in the decrease of overtreatment from 61 patients (53%) to 8 patients (19%). From 21 patients (28%) who would stay undertreated, 18 patients would reveal the PSA progression at day 90 while only 2 patients would stay undertreated to late appearance of BCR after 39 and 48 months. Only 1 out of 16 patients who presented at day 30 with PSA ≤0.01 ng/ml developed BCR during the follow-up.

## Discussion

The removal of the entire prostate gland is a primary goal of radical prostatectomy. A positive surgical margin is defined as the presence of tumor at the inked surface of the resected specimen and as such implies inadequate cancer clearance. Despite advances in surgical techniques and the significant stage shift in newly diagnosed prostate cancer in the last decade, PSM are still reported in 11–38% of patients who have undergone RP [1, 2]. Comparable results were found in the present study, where PSM was diagnosed in 21% of patients after radical prostatectomy.

Although the true impact of a PSM remains controversial, many authorities agree it presents a significant risk of biochemical and subsequent clinical relapse. Authors from Johns Hopkins Hospital reported 79% of men with negative margins were progression-free over a 10 year period compared with 55% of those with positive margins [18]. Others have demonstrated similar findings [3, 19]. However not every patient will suffer eventual disease recurrence and the policy of adjuvant radiotherapy could result in considerable over-treatment. In the observation arms of large randomized trials where high-risk patients were involved, up to 52% did not show a BCR during the follow-up [20]. However, some results of these trials are conflicting due to the differences in the number and type of adverse pathologic characteristics included [21]. It has been demonstrated that in daily clinical practice, positive surgical margins present an independent predictor of secondary prostate cancer treatment [7]. Therefore, in our study we based the analysis only on a cohort of patients with the diagnosis of PSM and we found out that potential adjuvant radiation would result in overtreatment in the majority of the patients (53%).

Several explanations may explain why a positive margin is not always associated with the recurrence of cancer. It has been proposed that ischemia and fibrosis as a consequence of the surgery may destroy small areas of residual carcinoma [22]. Possible disruption of additional tissue covering cancer cells during all the handling of the specimen by surgeons, nurses and pathologists may result in inadvertent damage leading to the false impression of PSM. Finally the experience of the reading pathologist and the type of classification may influence the accuracy of surgical margin diagnostics. Nevertheless, the question remains how to reliably identify patients with residual prostate cancer cells in the surgical bed who would be the best candidates for immediate adjuvant treatment.

The ability of imaging modalities in the staging process of prostate cancer remains limited. Current imaging devices are not endowed with sufficient resolution to detect extraprostatic extension, which is often microscopic [8]. Several groups have highlighted potential prognostic value of additional pathological factors such as the location, extent and number of positive margins [19, 23]. However, the subclassification of positive margins has not been standardized and there is no general consensus in the literature on how specific PSM-associated prognostic variables influence BCR or assist in clinical decision-making [2]. Intraoperative frozen section biopsy has been recommended in radical prostatectomy. However, routine use of frozen section on all cases to be "sure" of getting negative margins has not fulfilled its dream [9, 10]. A number of authors have described prognostic significance of PSM after adjusting for other clinical and pathological variables. The Johns Hopkins group demonstrated the effect of Gleason grade on outcome in men with positive margins. They reported positive surgical margins had no impact on 10 y probability of biochemical recurrence in men with Gleason score less than 7 [24]. Conversely, in our series of men with PSM, we have not noted any impact of age, Gleason score and/or the extraprostatic extension of the prostate cancer on the frequency of BCR. Ultrasensitive PSA assessed early after the operation was the only variable predicting the disease progression.

With removal of all the prostate cancer tissue, the serum PSA should rapidly decline to zero, or at least very close to zero within 2 to 6 weeks following radical prostatectomy. However, general recommendation advises to perform the first PSA check at 3 months after the surgery, although a significant decline in PSA and even the PSA nadir may be reached a few weeks prior to this point [11]. These results prompted us to investigate the PSA kinetics early after the surgery, starting with the first measurement at day 14. Our data demonstrate that PSA at day 14 is not offering valuable prognostic information regarding the outcome of the surgery. However, as time from surgery increased, the predictive power of ultrasensitive PSA measurements increased. For example, the calculated AUR for day 30 and day 60 were 74% and 84%, respectively. Serum PSA at early moments after the surgery may be influenced by the clearance of PSA, which was produced by the tissue of ablated prostate. This is compatible with our finding that preoperative PSA correlated only with postoperative PSA at day 14 and at day 30.

It has been shown that retained benign prostatic elements are an unlikely source of elevated PSA levels in men who have undergone radical prostatectomy. In a study of Godoy et al. only 0.3% out of 331 men with low-risk prostate cancer had developed a measurable PSA level after radical prostatectomy [16]. Odisho et al. have reviewed 274 patients with benign glandular tissue at the surgical margin after radical prostatectomy. They concluded that this finding was not associated with postoperative elevation of PSA [15]. Thus, it seems that the only significant source of PSA after radical prostatectomy may be retained malignant cells and PSA kinetics early after the surgery are key to identification of patients with prostate cancer who have received failed prior therapy. In a series of Hong et al. there were 106 (27.6%) subjects who had a positive surgical margin after radical prostatectomy. Among these men, 45 patients, who showed undetectable ultrasensitive PSA nadir (PSA < 0.001 ng/ml) during the postoperative follow-up, demonstrated a significantly higher rate of 3-year biochemical recurrence-free survival compared with 61 men who did not (94.1% vs 57.1%, P < 0.001). In line with these results we observed that only 1 of 16 patients who reached PSA ≤0.01 ng/ml at the first month after the surgery experienced BCR during the follow-up.

This study has several potential limitations. These include limitations inherent to any retrospective study. There were no strict criteria for subjecting the patients with PSM after radical prostatectomy to adjuvant therapy during the whole study period. It may be argued that by doing so some patients with PSM who received adjuvant treatment were at high risk of the recurrence and they were excluded from the analysis. However, the comparison of clinicopathological characteristics did not reveal any significant difference between studied group of patients and those who underwent immediate secondary therapy after the surgery. Another limitation of our study was the relatively short mean follow-up of 31 months. Several investigators have reported that most biochemical recurrences are detected within the first 3 years after radical prostatectomy [25]. Nevertheless, presented results should be analyzed with caution, as patients with BCR do not necessarily share the same long-term cancer outcomes. Our results will need re-evaluation as our follow-up matures to yield meaningful data on cancer specific survival, as the most relevant endpoint.

## Conclusions

The present study provides insights into the role ultrasensitive serum PSA measurements plays in determining who will develop BCR after radical prostatectomy and, such as, be candidates for secondary treatment. The kinetics of postoperative PSA decline may allow better stratification of patients who would benefit from immediate radiation therapy.

## Abbreviations

PSM: Positive surgical margins
RP: Radical prostatectomy
PSA: Prostate-specific antigen
BCR: Biochemical recurrence
AUC: Area under the curve
HR: Hazard ratio
ROC: Receiver operating characteristic curve.
